# Tissue resident memory T cells in the human conjunctiva and immune signatures in human dry eye disease

**DOI:** 10.1038/srep45312

**Published:** 2017-03-27

**Authors:** Tanima Bose, Ryan Lee, Aihua Hou, Louis Tong, K. George Chandy

**Affiliations:** 1 Laboratory of Molecular Physiology, Infection and Immunity Theme, Lee Kong Chian School of Medicine, Nanyang Technological University, 59 Nanyang Drive, 636921, Singapore; 2 Singapore Eye Research Institute, 20 College Road, 169856, Singapore; 3 Duke-NUS Medical School, 8 College Road, 169857, Singapore; 4 Singapore National Eye Center, 11, Third Hospital Avenue, 168751, Singapore; 5 Yong Loo Lin School of Medicine, 1E Kent Ridge Road Level 7, NUHS Tower Block, 119228, Singapore.

## Abstract

Non-recirculating resident memory (T_RM_) and recirculating T cells mount vigorous immune responses to both self and foreign antigens in barrier tissues like the skin, lung and gastrointestinal tract. Using impression cytology followed by flow cytometry we identified two T_RM_ subsets and four recirculating T-subsets in the healthy human ocular surface. In dry eye disease, principal component analysis (PCA) revealed two clusters of patients with distinct T-cell signatures. Increased conjunctival central memory and naïve T cells characterized Cluster-1 patients, and increased CD8^+^ T_RM_s and CD4^+^ recirculating memory T cells characterized Cluster-2 patients. Interestingly these T-cell signatures are associated with different clinical features: the first signature correlated with increased ocular redness, and the second with reduced tear break up times. These findings open the door to immune-based characterization of dry eye disease and T-subset specific immunotherapies to suppress T-subsets involved in disease. They may also help with patient stratification during clinical trials of immunomodulators.

Infections or antigenic challenge cause naïve T cells to differentiate into distinct memory T cell populations that are distinguished by their expression of the chemokine receptor CCR7 and the tyrosine phosphatase CD45 (T_CM_: central memory T cells; T_EM_: effector memory T cells: T_EMRA_: effector memory T cells that have reacquired expression of CD45RA and lost CD45RO) (Fig. 1A)^1^. Each of these subsets can give rise to effector cells when challenged by their cognate antigen. In barrier tissues such as the skin, lung, gastrointestinal tract and genitourinary tract, T_EM_ and T_EMRA_ cells differentiate further into non-recirculating tissue resident memory T cells (T_RM_), which persist for long periods in these tissues in the absence of antigens and provide immune protection^2^,^3^,^4^,^5^,^6^,^7^,^8^,^9^,^10^,^11^. The change from recirculating T_EM_/T_EMRA_ cells to non-recirculating T_RM_ cells involves the acquisition of the type-C lectin CD69 and the integrin (αEβ7) CD103 (Fig. 2A)^2^,^3^,^4^,^5^,^6^,^7^,^8^,^9^,^10^,^11^. Two subsets of T_RM_ cells have been described based on the expression of CD69 and CD103: CD69^+^CD103^−^ and CD69^+^CD103^+^ subsets^2^,^3^,^4^,^5^,^6^,^7^,^8^,^9^,^10^,^11^. Both T_RM_-subsets are potent effectors, but CD69^+^CD103^+^ T_RM_s exhibit limited proliferative capacity compared to CD69^+^CD103^−^ T_RM_s^4^,^5^. The distribution of these T cell subsets has not been determined in the human ocular surface, a barrier tissue that protects sensitive internal structures responsible for vision^12^.

The ocular surface consists of the cornea, the conjunctiva, the lacrimal gland and the lacrimal drainage system, and meibomian glands. The mucosa covering the avascular corneal surface at the visual axis contains mainly myeloid and dendritic cells, while the conjunctiva over the remainder of the ocular surface also contains lymphatics, diffuse and aggregated lymphocytes^13^,^14^. While T cell subsets have been described in the conjunctiva^13^, the relative proportions of T_RM_ and recirculating T cells at this barrier surface have not been studied in humans or animal models. Such information could provide insights into ocular surface inflammation.

The most common and prominent manifestation of ocular surface inflammation is dry eye disease (DED). DED is a heterogeneous group of diseases, with overlapping but distinct clinical features. Symptoms of discomfort and visual disturbance as a result of tear instability, increased osmolarity of the tear film, and inflammation of the ocular surface characterize DED. The prevalence of DED increases with age, ranging from 5% in young adults to as much as 35% in people of 50 years and older^15^,^16^,^17^,^18^,^19^,^20^,^21^,^22^. It develops due to stress, autoimmune diseases (rheumatoid arthritis, systemic lupus erythematosus, Sjogren’s syndrome), chemical injury, blepharitis, infections, meibomian gland dysfunction and allergies, and more devastating conditions such as Steven Johnson syndrome and graft-versus-host disease^15^,^16^,^17^,^18^,^19^,^20^,^21^,^22^.

Many reports document the pathogenic role of T cells in animal models of DED^23^,^24^. The disease can be induced in animal models by the transfer of pathogenic T cells^25^. Animals with defects in regulatory T cells exhibit more severe DED^26^,^27^. The importance of T cells in human DED is underscored by the efficacy of cyclosporine, a T cell immunosuppressant that is a cornerstone of DED therapy^28^,^29^,^30^. Here, we use impression cytology and flow cytometry to determine the distribution of T_RM_ and recirculating T cell subsets at the ocular surface in healthy humans and in patients with dry eye disease. We demonstrate that the human ocular surface is protected by two subsets of T_RM_ cells and four subsets of recirculating T cells. We describe two immunological signatures in DED patients along with distinguishing clinical profiles. Our results raise the possibility of using the immune signatures and related clinical findings to stratify patients during clinical trials of immunomodulators. It also suggests the feasibility of developing an immune-based classification of DED and could lead to novel immunotherapies that target specific immune signatures to complement current broadly immunosuppressive therapies.

## Results

### CD8^+^ tissue resident memory T cells predominate in the normal human ocular surface

**Figure 1:**
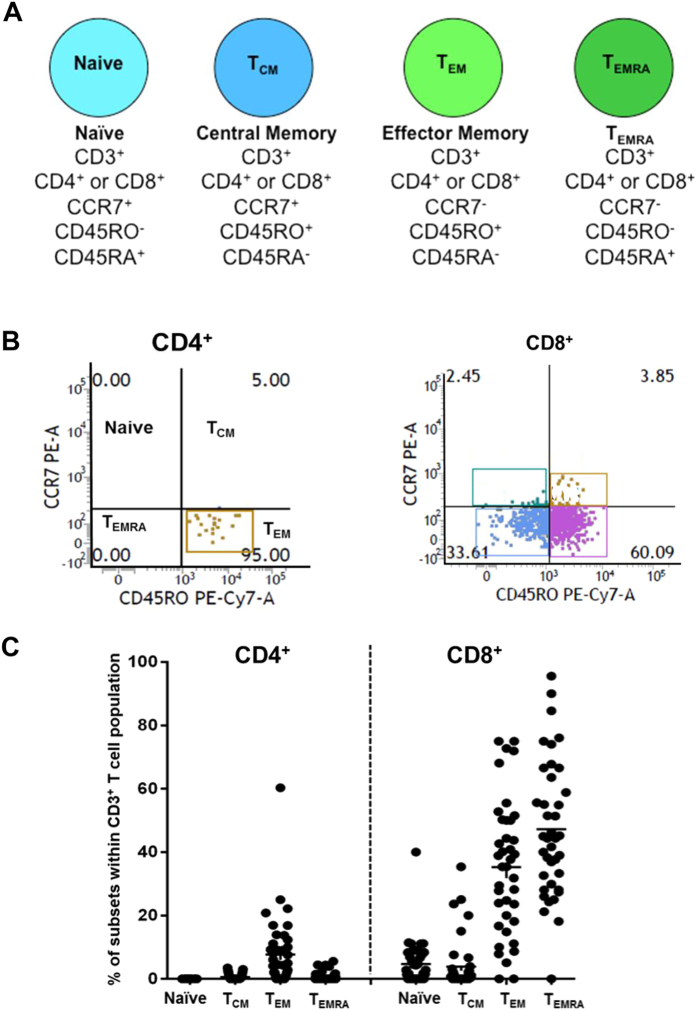
T-cell surface markers in normal ocular surface. (**A**) Cell surface markers for naïve, T_CM_, T_EM_ and T_EMRA_ subsets used in our studies. (**B**) Flow cytometry showing proportion of naïve, T_CM_, T_EM_ and T_EMRA_ subsets within CD4^+^ and CD8^+^ pools (*left and right panels)* in the nomal human conjunctiva. (**C**) Proportion of conjunctival naïve, T_CM_, T_EM_ and T_EMRA_ subsets in CD4^+^ and CD8^+^ T cell pools expressed as a percentage of the total number of CD3^+^ T cells in healthy human conjunctiva. Each data point represents a separate individual; mean ± SEM shown.

We selected 39 healthy controls based on the absence of history of DED symptoms in the preceding 3 weeks determined by the SPEED (Standard Patient Evaluation of Eye Dryness) questionnaire, and normal scores on the Schirmer’s Test and non-invasive tear breakup time (NI-TBUT) (Supplementary Table S1, Supplementary Figure S1). Four impressions were obtained per individual, two impressions from each eye. Cells were isolated from the membrane and flow cytometry used to analyse T cell subsets (Fig. 1A). CD3^+^ T cells were gated out and live AAD^−^ cells selected. The total number of CD3^+^ T cells obtained from each patient varied from ~300–1800. We determined the proportions of CD4^+^ and CD8^+^ T cells within the CD3^+^ pool (Fig. 1B). We then measured the proportions of naïve, T_CM_, T_EM_ and T_EMRA_ T cells within the CD4^+^ and CD8^+^ pools. CD8^+^ T cells accounted for ~90% of CD3^+^ T cells in the normal conjunctiva (Fig. 1C). Of CD8^+^ T cells, ~80% were CCR7^−^ T_EM_ and T_EMRA_ cells, and a minority were CCR7^+^ naïve T and T_CM_ cells (Fig. 1C). Only 10% were CD4^+^ T cells and these were almost entirely T_EM_ cells (Fig. 1C). Our results are consistent with an earlier study, which reported a preponderance of CD8^+^ T_EM_ cells in the ocular surfaces of a cohort of human volunteers in the UK^13^.

**Figure 2:**
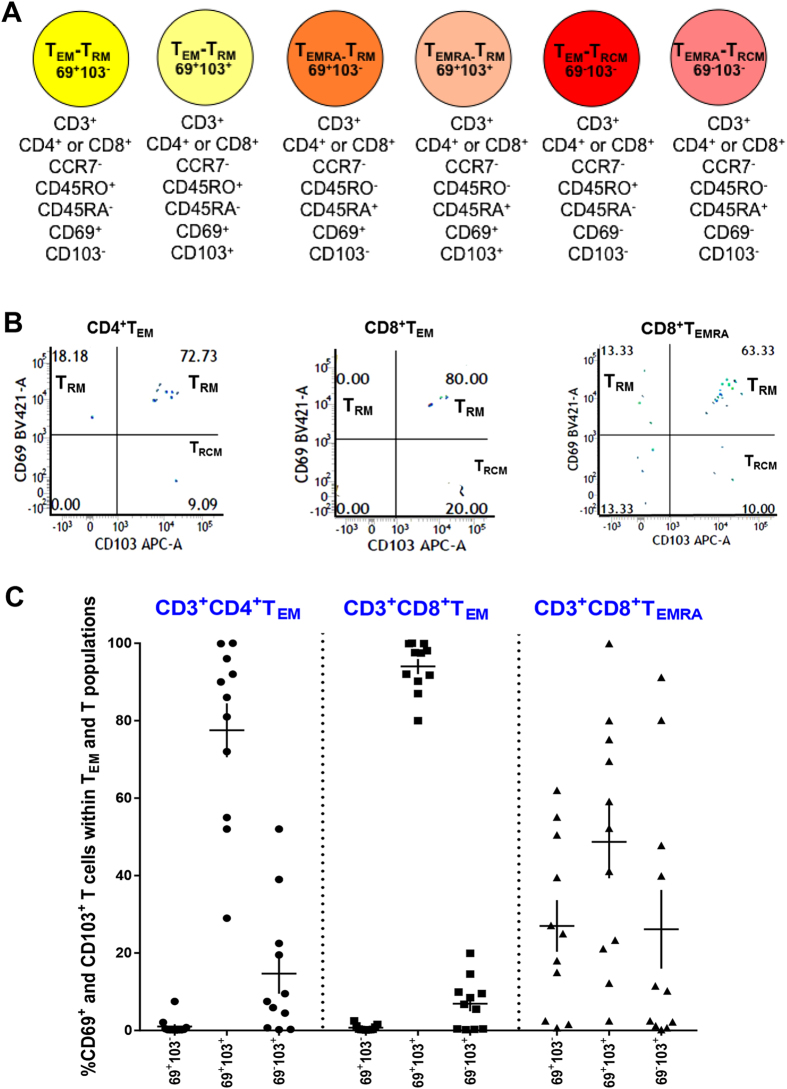
Tissue resident memory T cells predominate in the human ocular surface. (**A**) Cell surface markers for T_RM_ and T_RCM_ subsets used in our studies. (**B**) Flow cytometry showing proportion of CD69^+^CD103^−^T_RM_, CD69^+^CD103^+^ T_RM_ and T_RCM_ in CD4^+^ T_EM_, CD8^+^ T_EM_ and CD8^+^ T_EMRA_ subsets. (**C**) Proportion of conjunctival T_RM_s (CD69^+^CD103^−^, CD69^+^CD103^+^), T_RCM_ (CD69^−^CD103^+^) subsets within T_EM_ and T_EMRA_ pools in healthy controls. The different T_RM_ populations in each T_EM_/T_EMRA_ pool do not add up to 100% because we did not include CD69^−^CD103^−^ T cells in the figure. Each data point represents a separate individual; mean ± SEM shown.

We next measured the proportion of the two non-recirculating resident memory T_RM_ subsets (CD69^+^CD103^−^, CD69^+^CD103^+^) within the cell populations above (Fig. 2A). A third subset (T_RCM_) expresses CD103 but lacks CD69 (Fig. 2A). In rodents, this subset has been reported to be recirculating memory T cells that migrate from epithelial tissues via draining lymph nodes and the circulation to distant epithelial sites of inflammation^31^. We used coordinate analysis of CD69 and CD103 expression to quantify all three memory subsets within the CD8^+^ and CD4^+^ T_EM_/T_EMRA_ pools in conjunctivas of 13 healthy controls. Representative FACS profiles are shown in Fig. 2B. In both CD8^+^ and CD4^+^ T_EM_/T_EMRA_ subsets, CD69^+^CD103^+^ T_RM_s accounted for 70–80%, and CD69^+^CD103^−^ T_RM_s and T_RCM_s each accounted for 5–10% (Fig. 2C). Since the chemokine receptors CCR6 and CXCR3 are necessary for T cell-mediated ocular surface inflammation in experimental DED^32^, we measured T cell subsets expressing these receptors in our subjects. The majority of T cells were CXCR3^+^CCR6^−^, with CXCR3^−^CCR6^+^ and CXCR3^+^CCR6^+^ T cells being less abundant (Supplementary Figure S2). In summary, CD8^+^ T_EM_/T_EMRA_ cells predominate in the human ocular surface. Of these the majority are CD69^+^CD103^+^ T_RM_s. Four recirculating T cells subsets are also present: naïve, T_CM_, T_EM_/T_EMRA_ and T_RCM_s.

### Two immune signatures in patients with dry eye disease

We identified another 52 participants that satisfied our clinical criteria (see below) of having DED. Since DED is multifactorial, we were interested to know if the disease is heterogeneous in terms of conjunctival T cell composition. T cells in the conjunctiva of these 52 DED patients were therefore immunophenotyped to identify proportions of T subsets (naïve, T_CM_, T_EM_, T_EMRA_, T_RM_s, T_RCM_) present during ocular surface inflammation. Patients with DED were selected based on presence of at least *two* of the following: complaints of dry eye symptoms >2 weeks duration determined by the SPEED questionnaire, and abnormal Schirmer’s Test and/or NI-TBUT score. In our participants, DED was due to idiopathic causes (n = 30), Sjogren’s syndrome (n = 5), graft-versus-host disease (n = 5), rheumatoid arthritis (n = 4), systemic lupus erythematosus (n = 2), glaucoma medications (n = 3), mixed connective tissue (n = 1), myelodysplasia (n = 1), and contact lens wear (n = 1) (Supplementary Tables S2 and S3). These patients were age-matched and gender-matched with the controls above (Supplementary Figure S1).

**Figure 3:**
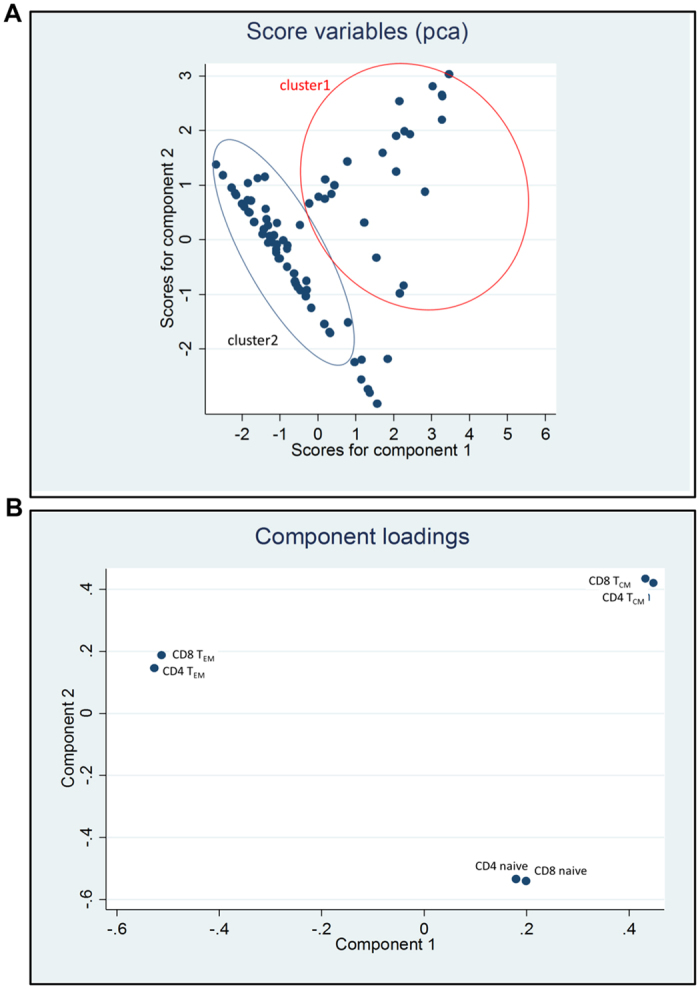
Principal Component Analysis reveals two T cell signatures in dry eye disease. (**A**) 2D Scatter diagram showing first 2 principal components. (**B**) Loading-plot showing the first principal component is largely determined by the ratio of T_CM_ to T_EM_, a higher ratio will shift the point to the right of the horizontal axis, whereas a lower ratio will be represented on the left.

**Figure 4:**
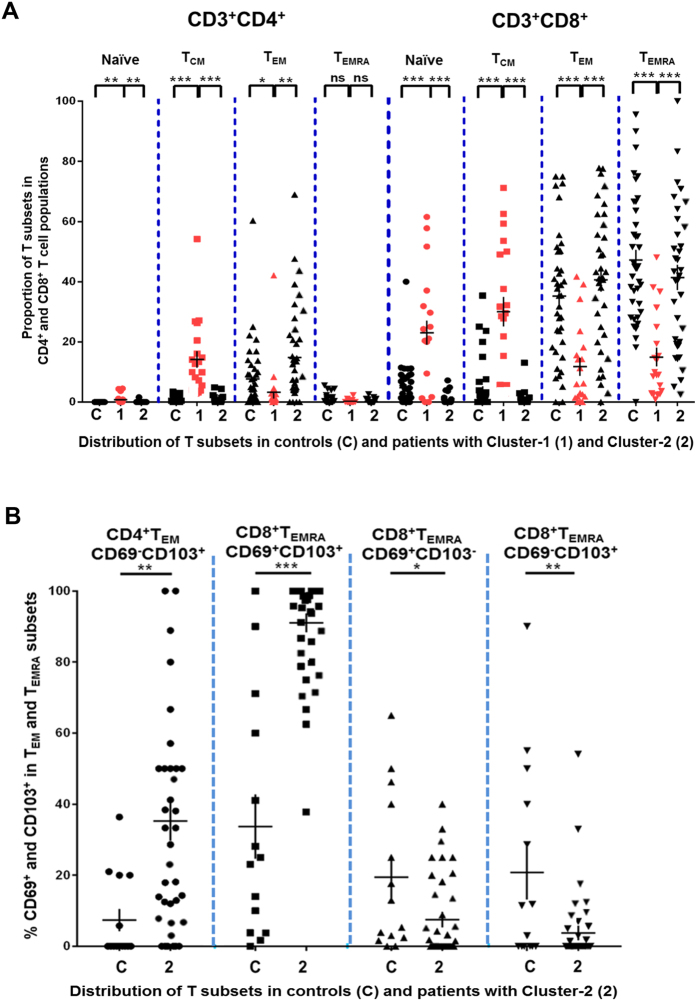
Two T cell signatures in dry eye disease. (**A**) Conjunctival CD4^+^ and CD8^+^ naïve, T_CM_, T_EM_ and T_EMRA_ subsets in controls (C) and Cluster-1 patients (1) and Cluster-2 patients (2). Each data point represents a separate individual; mean ± SEM shown. (**B**) Conjunctival T_RM_, T_RCM_ subsets within CD4^+^T_EM_ and CD8^+^ T_EMRA_ pools in controls (C) and Cluster-2 patients (2). Each data point represents a separate individual; mean ± SEM shown.

We performed principal component analysis of DED patients based on the proportion of different T cell subsets (CD4^+^ and CD8^+^ naïve, T_CM_ and T_EM_). Two clusters of patients were revealed when we plotted the first 2 principal components (Fig. 3A,B). Differences between these clusters were attributed largely to the first principal component (horizontal axis in Fig. 3B). We classified DED into two groups: 16 patients in Cluster-1 (red circle on right in Fig. 3A) and 36 patients in Cluster-2 (blue circle on left in Fig. 3A). Cluster-1 patients exhibited increased conjunctival CD4^+^ and CD8^+^ naïve and T_CM_ cells and decreased CD4^+^ and CD8^+^ T_EM_ and T_EMRA_ cells compared to Cluster-2 patients (Fig. 4A). We refer to this T cell subset pattern in DED as *T-cell signature-1*. Cluster-2 patients had increased CD3^+^ T_EM_ and T_EMRA_ cells compared to Cluster-1 patients (Fig. 4B). We refer to this T cell subset pattern as *T-cell signature-2*. The absolute numbers of T cells are shown in Supplementary Tables S4 and S5. In Cluster-1 patients CD3^+^ T_CM_ and naïve T cells (T_CM_ = 588 ± 73; naïve = 180 ± 10) were higher than in Cluster-2 patients (T_CM_ = 66 ± 12; naïve = 44 ± 12) (T_CM_ = *P* < 0.0001; naïve = *P* < 0.01). In Cluster-2 patients, the absolute numbers of CD3^+^ T_EM_ and T_EMRA_ cells (T_EM_ = 853 ± 10; T_EMRA_ = 617 ± 12) were higher than in Cluster-1 patients (T_EM_ = 176 ± 10; T_EMRA_ = 162 ± 12) (for both T_EM_ and T_EMRA_ subsets = *P* < 0.0001).

### Comparison of T cell signatures in dry eye disease versus controls

A logical next step is to determine differences between the two T cell signatures in DED with the controls evaluated in Figs 1 and 2. Using *t-tests*, we found that Cluster-1 patients had increased conjunctival CD4^+^ and CD8^+^ naïve and T_CM_ cells, and decreased CD4^+^ and CD8^+^ T_EM_ and T_EMRA_ cells compared to controls (Fig. 4A). Cluster-2 patients had increased CD4^+^CD69^−^CD103^+^ T_EM_-T_RCM_ and CD8^+^CD69^+^CD103^+^ T_EMRA_-T_RM_, and decreased CD8^+^CD69^+^CD103^−^ T_EMRA_-T_RM_ and CD8^+^CD69^−^CD103^+^ T_EMRA_-T_RCM_ compared to controls (Fig. 4B). The absolute numbers of T cells are shown in Supplementary Tables S4 and S5. In Cluster-1 patients, the absolute numbers of CD3^+^ T_CM_ and naïve T cells (T_CM_ = 588 ± 73; naïve = 180 ± 10) was higher than in controls (T_CM_ = 73 ± 12; naïve = 58 ± 12) (for both T_CM_ and naïve = *P* < 0.0001). In Cluster-2 patients, the absolute numbers of CD3^+^ T_EM_ and CD3^+^ T_EMRA_ patients (T_EM_ = 853 ± 10; T_EMRA_ = 617 ± 12) were similar to that in controls (T_EM_ = 632 ± 12; T_EMRA_ = 691 ± 12) (Supplementary Table S4). However, Cluster-2 patients had higher absolute numbers of CD8^+^CD69^+^CD103^+^ T_EMRA_-T_RM_ (528 ± 8) and CD4^+^CD69^−^CD103^+^T_EM_-T_RCM_ (101 ± 1) than controls (CD8^+^CD69^+^CD103^+^ T_EMRA_-T_RM_ = 251 ± 6; CD4^+^CD69^−^CD103^+^T_EM_-T_RCM_ = 9 ± 2) (CD8^+^CD69^+^CD103^+^ T_EMRA_-T_RM_ = *P* < 0.0001; CD4^+^CD69^−^CD103^+^T_EM_-T_RCM_ = *P* < 0.01). Cluster-2 patients had lower absolute numbers of CD8^+^CD69^+^CD103^−^T_EMRA_-T_RM_ (41 ± 2) and CD8^+^CD69^−^CD103^+^ T_EMRA_-T_RCM_ (16 ± 2) compared to controls (CD8^+^CD69^+^CD103^−^T_EMRA_-T_RM_ = 132 ± 2; CD8^+^CD69^−^CD103^+^ T_EMRA_-T_RCM_ = 146 ± 2) (CD8^+^CD69^+^CD103^−^T_EMRA_-T_RM_ = *P* < 0.05; CD8^+^CD69^−^CD103^+^ T_EMRA_-T_RCM_ = *P* < 0.01) (Supplementary Table S4 and S5). These results indicate that the proportions of particular T_EM_ and T_EMRA_ subsets are altered in Cluster-2 patients, although the overall number of terminally differentiated CCR7^−^ T cells in these patients is the same as in controls. We did not find any difference in the proportions of T cells expressing the chemokine receptors CXCR3 or CCR6 in Cluster-1 or Cluster-2 patients compared to each other or to controls (data not shown). In summary, we identified two clusters of DED patients with distinct T cell immune signatures which are also significantly different from controls.

### The two ocular T-cell signatures in dry eye patients correlate with different clinical features

To understand the clinical implications of the two T cell signatures in DED, we examined whether Cluster-1 and Cluster-2 patients derived from principal component analysis demonstrated any differences in clinical phenotypes.

**Figure 5:**
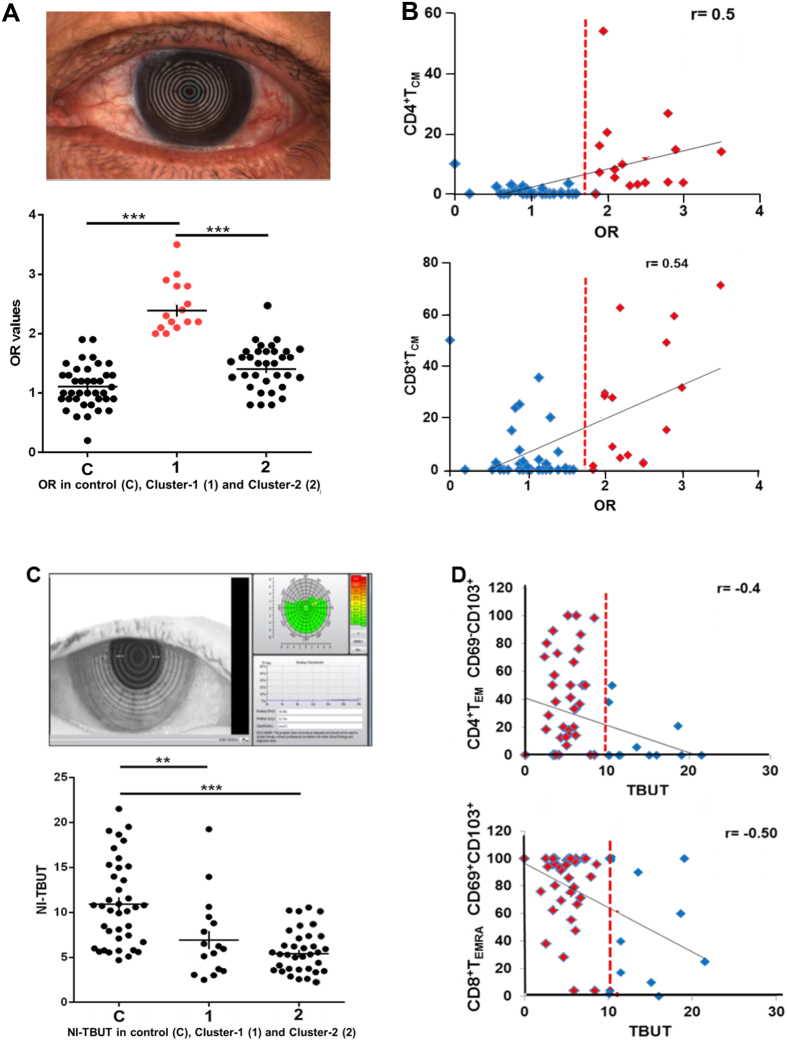
Correlation of two T cell signatures with clinical parameters. (**A**) *Upper panel*: ocular redness; *Lower panel*: Ocular redness (OR) scores in controls (C) Cluster-1 patients with T cell signature-1 (1) and Cluster-2 patients with T cell signature-2 (2). (**B**) OR scores show a positive correlation with CD4^+^ T_CM_ and CD8^+^ T_CM;_ controls (blue); Cluster-1 patients with T cell signature 1 (red); dotted red line indicates OR 1.9. Pearson’s correlation coefficient was calculated for 55 subjects. Two-tailed P < 0.0001 for both the correlations. (**C**) U*pper panel*: Picture of ocular surface obtained with an Oculus Keratograph 5 M; *Lower panel*: NI-TBUT in controls (C) and Cluster-1 patients with immune signature-1 (1) and Cluster-2 patients (2). (**D**) NI-TBUT correlates with CD4^+^CD69^−^CD103^+^T_EM_-T_RCM_ and CD8^+^CD69^+^CD103^+^ T_EMRA_-T_RM_; controls (blue); Cluster-2 patients with T cell signature-2 (red); dotted red line indicates NI-TBUT = 10 s. Pearson’s correlation coefficient was calculated for 49 subjects. Two-tailed *P* < 0.0001.

When *t-tests* were performed, we found that ocular redness (OR)^33^ was significantly increased in Cluster-1 compared to Cluster-2 patients (*P* < 0.001) (Fig. 5A, Table 1). OR arises as a consequence of reactive dilation of conjunctival blood vessels (conjunctival hyperemia) and is a frequent response to diverse pathologic stimuli^33^,^34^,^35^. When all controls and patients were considered, we detected a positive linear correlation between OR scores and conjunctival CD4^+^ T_CM_ (r = 0.5; *P* < 0.0001) and CD8^+^ T_CM_ (r = 0.54; *P* < 0.0001), and an inverse correlation with conjunctival CD8^+^ T_EM_ (r = −0.4; *P* = 0.0057) and T_EMRA_ cells (r = −0.5; *P* = 0.002) (Fig. 5B and Supplementary Fig. S3).

NI-TBUT assesses tear evaporation, spreading and elasticity of tears in DED. In Cluster-2 patients, shorter NI-TBUT is indicative of increased tear instability correlated with higher proportions of conjunctival CD4^+^ T_EM_-T_RCM_ (r = −0.4; *P* < 0.0001) and CD8^+^ T_EMRA_-T_RM_s (r = −0.5; *P* < 0.0001) (Fig. 5D, Supplementary Figure S4).

When the data were re-analyzed only with patients with idiopathic DED, we found the same two clusters and associated clinical findings. This indicates that the differences in immune signatures are not likely to be due to differences in specific etiology of DED in the two clusters (specific etiologies provided in Supplementary Tables S2 and S3).

**Figure 6:**
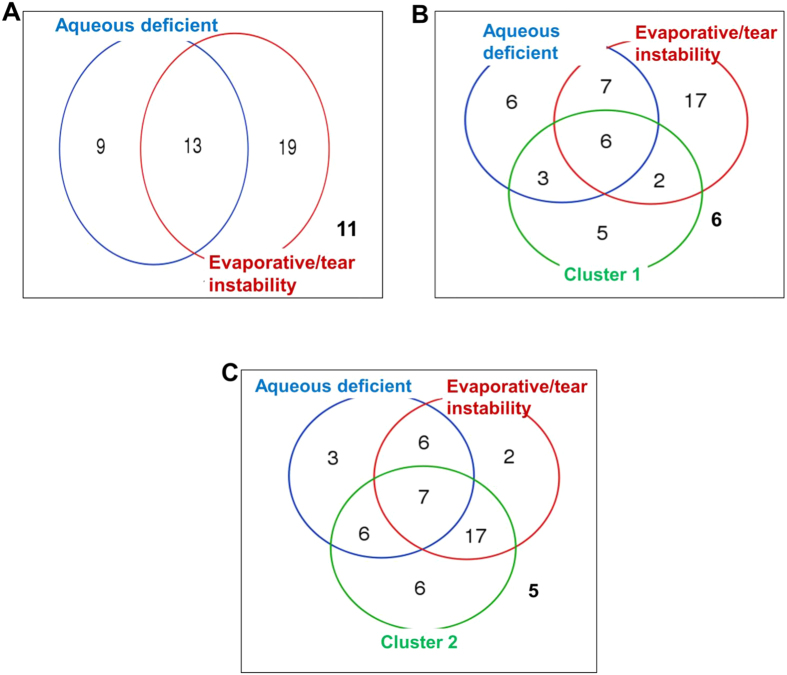
Venn Diagram analysis. (**A**) Proportion of DED patients (n = 52) with aqueous deficiency dry eye (Schirmer test <5 mm) or evaporative dry eye (NI-TBUT <6 s). (**B**,**C**) Proportion of Cluster-1 or Cluster-2 dry eye patients with aqueous deficiency dry eye or evaporative dry eye, respectively. The subject numbers analyzed for this diagram was 52. In all three panels, the numbers outside the circles are patients who did not qualify as aqueous, or evaporative or mixed. Venn diagram analysis was performed using online software at the following URL: http://bioinformatics.psb.ugent.be/webtools/Venn/.

Dry eye disease is commonly classified into two clinical subtypes based on the Schirmer and NI-TBUT tests^21^. “Aqueous deficiency dry eye” is defined as a Schirmer test <5 mm, and “evaporative or tear deficiency DED” is defined as NI-TBUT <6 secs. In this study, 17% (9/52) of DED patients had pure aqueous deficiency DED (Fig. 6A), 37% (19/52) had pure evaporative/tear instability DED, 25% (13/52) had combined aqueous deficiency and evaporative DED, and 21% (11/52) did not qualify as aqueous or evaporative or mixed subtypes based on their average NI-TBUT and Schirmer values in the right and left eyes (Fig. 6A). We used Venn diagram analysis to examine the overlap between Cluster-1 and Cluster-2 patients and these clinical subtypes of DED (Fig. 6A–C). There was a higher proportion of pure evaporative/tear instability sub-type patients in Cluster-2 (P = 0.027; Table 1, Fig. 6C). There were similar proportions of aqueous deficiency DED subtype and mixed cases in both clusters (Table 1, Fig. 6B and C). Our data provide additional non-redundant information about dry eye disease that might complement and facilitate patient stratification.

In summary, Table 1 shows that conjunctival redness (OR measured by an Oculus K5M)^33^ was significantly higher in Cluster-1 compared to Cluster-2 patients (P < 0.001 in all comparisons). Cluster-2 had a higher proportion of females compared to Cluster-1 (Table 1). All other parameters were identical between the clusters (Table 1). Male gender was associated with a higher OR (P = 0.016), so gender could potentially confound the relationship between the T-cell clusters and OR. In a linear regression model with OR as a dependent variable and T-cell cluster and gender status as independent variables, T-cell clustering remained significant (P < 0.001) while gender was no longer significant (P = 0.5). Finally, the proportion of pure evaporative/tear instability sub-type patients was significantly higher in Cluster-2 (P = 0.027).

## Discussion

The ocular surface comprised of the cornea, conjunctiva, meibomian glands, and lacrimal gland and drainage system is a barrier tissue that protects the vision apparatus^12^. T lymphocytes have been described in the human and rodent conjunctiva^13^, and ocular surface T cells play a critical pathogenic role in DED, the commonest clinical manifestation of ocular surface inflammation^22^,^23^,^24^,^25^,^26^,^27^,^28^,^29^,^30^. Here, we have used impression cytology and flow cytometry to characterize recirculating (naïve, T_CM_, T_EM_, T_EMRA_, T_RCM_) and non-recirculating (T_RM_) T cell subsets (Figs 1A and 2A) in the healthy human ocular surface and in patients with DED.

**Figure 7:**
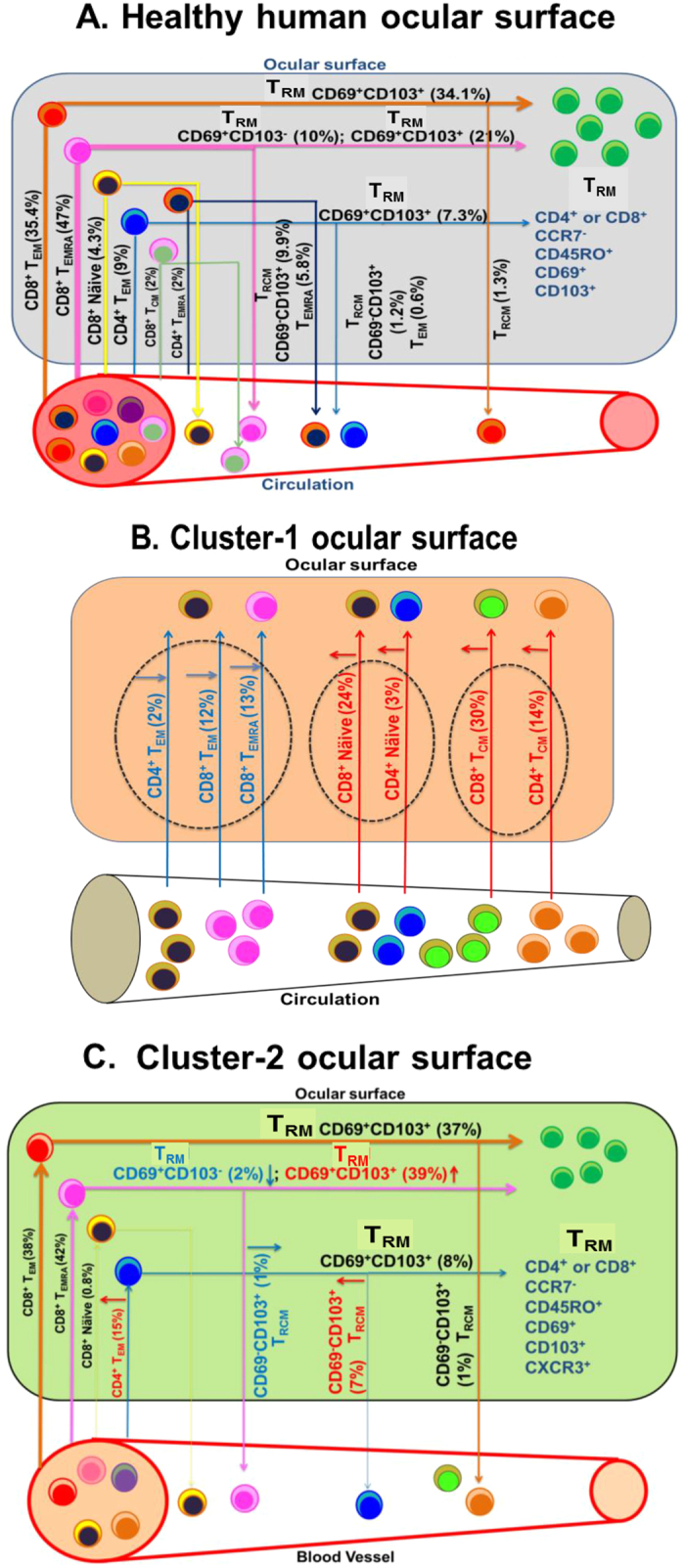
T cell subset patterns in the ocular surface in controls and cluster-1 and cluster -2 patients. (**A**) Schematic summarizing distribution of recirculating (naïve, T_CM_, T_EM_, T_EMRA_, T_RCM_) and non-recirculating (CD69^+^CD103^−^ T_RM_, CD69^+^CD103^+^ T_RM_) T-subsets in the healthy human ocular surface. Numbers indicate the proportion of each subset within the CD3^+^ T cell pool. (**B**) Schematic summarizing distribution of conjunctival T-subsets in Cluster-1 patients. Numbers indicate the proportion of each subset within the CD3^+^ T cell pool. (**C**) Schematic summarizing distribution of conjunctival T-subsets in Cluster-2 patients. Numbers indicate the proportion of each subset within the CD3^+^ T cell pool.

In the healthy human ocular surface, CD8^+^ T cells are the majority subset, and they are predominantly terminally differentiated CD8^+^ T_EM_ and T_EMRA_ cells (Figs 1D and 7A). CD4^+^ T cells are a minority, but they too are mainly T_EM_ and T_EMRA_ cells (Figs 1D and 7A). CD8^+^ and CD4^+^ T_EM_ and T_EMRA_ cells are preponderantly non-recirculating T_RM_s, the CD69^+^CD103^+^ T_RM_ subset being most abundant (Figs 2A and 7A). We also detect recirculating CCR7^+^ naïve and T_CM_ cells, and recirculating CCR7^−^CD69^−^CD103^−^ T_EM_, T_EMRA_ and T_RCM_ cells in the healthy human in ocular surface. This T cell-distribution is similar to that in the gastric lamina propria. In both the ocular surface and the stomach’s lamina propria CD8^+^CD69^+^CD103^+^ T_RM_s predominate and CD4^+^ T cells are a minority^10^. CD8^+^CD69^−^CD103^+^ T_RCM_ recirculating cells are also present in both tissues^10^, but have not been reported in other barrier tissues such as the human skin, non-inflamed human lung, and intestinal and cervical mucosa. In the human dermis and non-inflamed lung CD4^+^CD69^+^CD103^−^ T_RM_s predominate, while in the human epidermis, intestinal mucosa and cervical mucosa a mixture of CD4^+^ and CD8^+^ CD69^+^CD103^+^ T_RM_ cells are present^2^,^3^,^4^,^5^,^6^,^7^,^8^,^9^,^10^,^11^. We do not yet understand the reasons for the similarity of the T cell profile in the human conjunctiva and other tissues.

Ocular surface inflammation in DED can arise from multiple etiologies including autoimmunity (Sjogren’s syndrome, rheumatoid arthritis, systemic lupus erythematosus), blepharitis, and cicatrizing conjunctivitis. In experimental rodent DED, T cells are implicated in disease pathogenesis and adoptive transfer of pathogenic T cells is sufficient to impart DED in naïve recipients^23^,^24^,^25^,^26^,^27^,^32^,^36^,^37^. Although the T-cell immunosuppressant cyclosporine A is a cornerstone of DED therapy, the direct involvement of T cells in human DED is harder to demonstrate. Using immunohistochemistry on conjunctival biopsies, the involvement of CD4^+^ and CD8^+^ T cells in Sjogren and non-Sjogren DED patients has been reported^38^,^39^. More recently, impression cytology has been used to demonstrate the preponderance of mucosal-homing CD8^+^ T_EM_ cells in the conjunctiva of healthy individuals^40^.

To determine if DED causes changes in the profile of recirculating and non-recirculating T cells, we used impression cytology and flow cytometry to study T cell subsets in the ocular surface of 52 patients with DED and 29 age- and gender-matched controls (Tables S1–S3). PCA revealed two distinct T-cell signatures (Fig. 7B,C), which we discovered to be associated with particular clinical features. Cluster-1 patients had increased conjunctival CD8^+^ and CD4^+^ CCR7^+^ naïve and T_CM_ T cells that may have been recruited to the ocular surface. Recruitment of CCR7^+^ T cells to tissues is mediated by chemokines CCL19 and CCL21 that bind to the CCR7 receptor. Both chemokines are present in tear-duct associated lymphoid tissue, and expression of CCL21 is increased in inflamed cornea^41^,^42^. These chemokines may recruit naïve and T_CM_ T cells to the ocular surface in Cluster-1 patients. These patients also had objectively increased OR (a measure of conjunctival hyperemia quantified by an Oculus Keratograph) compared to controls and Cluster-2 patients. The positive correlation between conjunctival naïve and T_CM_ T cells and ocular redness suggests that the mechanisms underlying recruitment of CCR7^+^ T cells to the ocular surface and induction of conjunctival hyperemia may be linked. NI-TBUT (measure of tear stability) was reduced in Cluster-1 patients compared to controls, but NI-TBUT was not correlated with the numbers of naïve and T_CM_ T cells. We interpret this lack of correlation to mean that the recruitment of CCR7^−^ T cells to the ocular surface is not related to the processes involved in tear evaporation, spreading and elasticity of tears.

Cluster-2 patients had similar absolute numbers of conjunctival CD3^+^ T_EM_ and T_EMRA_ cells as controls, but the relative proportions of conjunctival CD8^+^CD69^+^CD103^+^ T_EMRA_-T_RM_ and CD4^+^CD69^−^CD103^+^T_EM_-T_RCM_ cells were increased compared to controls with a corresponding decrease in CD8^+^CD69^+^CD103^−^ T_EMRA_-T_RM_ and CD8^+^CD69^−^CD103^+^ T_EMRA_-T_RCM._ (*T cell signature-2*) (Fig. 7C). These patients exhibited reduced NI-TBUT compared to controls, with shorter NI-TBUT values correlating with higher proportions of conjunctival CD4^+^ T_EM_-T_RCM_ and CD8^+^ T_EMRA_-T_RM_s. The increased T_RM_s and reduced NI-TBUT suggest that a chronic inflammatory process in Cluster-2 patients may have damaged elements on the ocular surface responsible for tear stability. In support, chronic DED in a rodent model is principally mediated by T_EM_ Th17 cells^37^.

Corticosteroids and/or cyclosporine are cornerstones of immunotherapy in DED^28^,^29^,^30^. Corticosteroids, while effective, raise intraocular pressure and cause glaucoma and cataracts with prolonged usage, while cyclosporine causes burning of the eyes^28^,^29^,^30^. Both drugs are broadly immunosuppressive. Novel immunotherapies that are more targeted are necessary. Since Cluster-1 patients exhibit increased CCR7^+^ T cells on the ocular surface, inhibitors of CCR7 may have therapeutic benefit in these patients. Topical application of a blocking antibody against CCR7 has been reported to ameliorate ocular surface inflammation in an ovalbumin sensitization rodent model^42^,^43^,^44^. This therapeutic effect has been attributed to the suppression of CCR7^+^ dendritic cells, but could also be due to suppression of CCR7^+^ T cells. The K_Ca_3.1 potassium channel is another target that could be engaged to preferentially suppress naïve and T_CM_ T cells in Cluster-1 patients while sparing T_EM_, T_EMRA_ and T_RM_ cells^45^. Senicapoc, a selective K_Ca_3.1 blocker was shown to be safe in human trials for other indications^46^, and could possibly be used topically for DED therapy. Since Cluster-2 patients have an increase in conjunctival CD8^+^CD69^+^CD103^+^ T_EMRA_-T_RM_ and CD4^+^CD69^−^CD103^+^T_EM_-T_RCM_ cell, drugs that preferentially target these subsets may have therapeutic benefit in these patients. The K_v_1.3 potassium channel is widely regarded as a target for preferential suppression of CCR7^−^ T_EM_ and T_EMRA_ cells^45^, and a K_v_1.3 inhibitor is in human trials as a therapeutic for autoimmune diseases^47^. Ocular formulations of K_v_1.3 inhibitors may have used in the treatment of such patients. Alefacept, a drug that depletes effector memory T cells and evaluated in human clinical trials^48^,^49^, may also be useful as an ocular formulation for Cluster-2 patients.

Our results show that the two conjunctival T-cell signatures of DED patients are unique classifiers. The proportion of patients with the evaporative/tear instability DED clinical sub-type are in a significantly higher in Cluster-2. Participants of clinical trials with DED could be stratified or selectively recruited based on T-cell signature, particularly if the treatment strategy selectively targets T-cell subsets. In medical centres which do not provide impression cytology and/or cytometric studies, documentation of OR, a non-invasive test, may provide a clue to the T-cell signature type in a DED patient. In a dry eye patient, elevation of OR suggests the T-cell signature of Cluster-1, and a normal OR suggests a Cluster-2 signature. Futures studies are required to determine if the two *T cell signatures* have differential prognosis or natural history in DED.

The strength of our study includes a uniform assessment of different kinds of patients with DED with objective and validated clinical tools. The limitation of our study is the lack of longitudinal data and the heterogeneity in terms of systemic treatment of these patients. It is extremely difficult to standardize treatment in a cross-sectional study, and would only be possible if assessment is performed as part of an interventional study where a uniform therapy can be started on freshly diagnosed patients. Another limitation is that we evaluated impression membranes instead of biopsies. Although these are relatively non-invasive, they may show a predisposition towards more superficial intraepithelial T cells as opposed to stromal cells and follicular T cells, and therefore do not represent the entire T cell population in the ocular surface mucosa. However, impression cytology is a standard procedure used in various studies of ocular surface disease^13^,^40^.

In summary, the human ocular surface is protected by two subsets of T_RM_s and four subsets of recirculating T cells, a profile resembling gastric mucosa more than skin, lung, intestine or cervix. We identify two clusters of DED that are distinguished by their immunological signatures and clinical tests. The two DED patient clusters may represent two distinct disease subsets with differing clinical outcomes, or they may represent interchangeable ends of an immune spectrum depending on whether inflammation is acute or chronic. Treatment follow-up studies are required to distinguish between these two possibilities. Our findings highlight the feasibility of stratifying DED into distinct sub-types that will allow assessment of treatment responsiveness, clinical outcomes, disease evolution, and prognosis. Our findings also open the door to novel sub-type-targeted therapies, which will complement current therapies.

## Materials and Methods

### Ethics

Human ocular samples were collected from Singapore National Eye Centre. The SingHealth Centralized Institutional Review Board and the Nanyang Technological University Institutional Review Board, Singapore approved all studies described here. Tenets of the Declaration of Helsinki were adhered to. All patients who met the eligibility criteria at the dry eye clinic in Singapore National Eye Centre went through study briefing and were invited for screening. Written informed consent was obtained from enrolled participants by the clinical trial coordinator. The study was registered at the clinicaltrials.gov database (Singapore National Eye Centre). Impression samples were collected few hours after medical tests were performed.

### Non-invasive tear breakup time (NI-TBUT)

We used the Oculus K5M to measure NI-TBUT^50^,^51^. Using infrared monitoring of the tear film, the Oculus algorithm documents the time and site of tear film breakup^34^. NI-TBUT value <10 secs was considered indicative of dry eye disease symptoms.

### Ocular redness

We used the Oculus K5M to objectively assess bulbar and peri-limbal redness of the eye. We took a photo of the ocular surface under white light with the Oculus camera and analyzed by comparing with a standard grading system (JENVIS) ranging from 0 (no redness) to 4.0 (maximum). The analysis also provided separate measures of redness of the temporal and nasal conjunctiva^33^,^34^. The following variables were used in the analysis: temporal bulbar OR, nasal bulbar OR, bulbar OR, limbal OR, total conjunctival OR. We used average OR values of the two eyes, as well as worse eye OR between the 2 eyes for analyses. These different ways of computing OR were used in order to ensure robustness of the findings, as there is no single universal parameter or threshold that is currently agreed upon.

### Schirmer’s Test

We measured baseline tear secretion using the Schirmer’s Test as described^52^. A Schirmer’s value <5 mm was considered indicative of tear hypo-secretion.

### SPEED questionnaire

We used the Standard Patient Evaluation of Eye Dryness (SPEED) questionnaire to assess dryness, irritation, burning and fatigue. All patients with DED had high SPEED scores^53^.

### Impression cytology

Impression samples were collected from nasal and bulbar portions of the eye by EyePrim technology after performing clinical tests on the subjects. Cells were isolated after collection by continuous scraping with a pipette tip. After scraping, cells were washed two times with flow cytometry staining buffer (PBS with 0.05% BSA).

### Flow cytometry

Samples isolated by impression cytology were incubated with fluorescent antibodies for 20–30 min and washed twice with staining buffer before measurement with the BD FACSVerse flow cytometer (BD Bioscience, San Jose, CA). All antibodies were purchased from BD Bioscience: CD3 brilliant violet 510 (BV510) (UCHT1), CD4 allophycocyanine-H7 (APC-H7) (SK3), CD8-fluorescein isothiocyanate (FITC) (RPA-T8), CD197/ CCR7 phycoerythrin (PE) (3D12), CD45RO phycoerythrin-cyanine7 (PE-Cy7) (UCHL1), Cell viability solution (7-aminoactinomycin D, 7-AAD), CD69 APC (FN50), CD103 BV421 (Ber-ACT8).

### Statistical analysis

Graphs were generated and statistical analyses were performed in GraphPad Prism 6.0 (GraphPad, La Jolla, CA). Statistical tests were evaluated using Mann-Whitney unpaired *t-test* and all data are expressed as mean ± SEM if not stated otherwise. *P < 0.05; **P < 0.01; ***P < 0.001. Venn Diagram analysis was performed using online software with the following url link: http://bioinformatics.psb.ugent.be/webtools/Venn/.

## Additional Information

**How to cite this article:** Bose, T. *et al*. Tissue resident memory T cells in the human conjunctiva and immune signatures in human dry eye disease. *Sci. Rep.* **7**, 45312; doi: 10.1038/srep45312 (2017).

**Publisher's note:** Springer Nature remains neutral with regard to jurisdictional claims in published maps and institutional affiliations.

## Supplementary Material

Supplementary Figures and Tables

## Figures and Tables

**Table 1:** Clinical characteristics of dry eye patients in Cluster-1 and Cluster-2.

Characteristic	Cluster 1 (n = 16)	Cluster 2 (n = 36)	P value^†††^
**Age**			
*Mean* ± *SD*	63.0 ± 15.1	56.0 ± 15.9	0.151
*Median (min, max*)	66.0 (27.0, 82.0)	58.6 (22.7, 90.7)	
**Gender**			
Women % (n)	44 (7/16)	86 (31/36)	<0.005^**^
**Ocular redness**^**†**^			
*Mean* ± *SD*	2.4 ± 0.6	1.5 ± 0.5	<0.001^***^
*Median (min, max*)	2.2 (1.4,3.5)	1.5 (0.8,2.9)	
**NI-TBUT**^**††**^ **(sec)**			
*Mean* ± *SD*	7.8 ± 5.8	5.2 ± 2.5	0.120
*Median (min, max)*	6.2 (2.5, 25.0)	5.0 (2.6, 14.1)	
**Schirmer I test (mm)**			
*Mean* ± *SD*	8.4 ± 8.8	9.4 ± 11.4	0.749
*Median (min, max*)	4.5 (0.5, 31.0)	6.0 (0.0, 45.0)	
**Subtype dry eye**			
Aqueous deficient %(n)	19 (3/16)	17 (6/36)	1.000
Evaporative %(n)	13 (2/16)	47 (17/36)	0.027*
Mixed%(n)	38 (6/16)	19 (7/36)	0.184
**Systemic disease**			
Chronic GvHD (n)	3	3	0.357
Primary Sjogren syndrome (n)	0	5	0.308
Rheumatoid arthritis (n)	1	3	1.000
SLE (n)	0	3	0.544
Myelodysplasia (n)	1	0	0.308
Glaucoma (n)	2	2	0.578
Hypothyroidism (n)	1	0	0.308
Mixed connective tissue (n)	1	0	0.308
**Ocular surgery**			
(LASIK) (n)	0	1	1.000
**Contact lens wear**			
(n)	1	0	0.308
Idiopathic (n)	8	22	0.548

^†^Average temporal bulbar redness shown (measured by Oculus Keratograph 5 M).

^††^Non-invasive tear break up time (measured by Oculus Keratograph 5 M).

^†††^Two-tailed p value (either chi-square/Fisher exact test or *t-test*).

*p < 0.05, **p < 0.01, ***p < 0.001.
