# Supplementary material for: Tissue resident memory T cells in the human conjunctiva and immune signatures in human dry eye disease
Source: Sci Rep. 2017 Mar 27;7:45312. doi: 10.1038/srep45312 (PMC5366884; doi:10.1038/srep45312)
Supplement: Supplementary Figures and Tables [file srep45312-s1.doc]

**Supplementary Dataset**

**Title: Tissue resident memory T cells in the human conjunctiva and immune signature in human dry eye disease**

**Running title:** Resident memory and recirculating T subsets in dry eye disease

**Authors:** Tanima Bose^1^, Ryan Lee^2^, Aihua Hou^2^, Louis Tong^2,3.4.5^*, K. George Chandy^1^*

**Keywords:** Dry eye disease; ocular surface; Conjunctiva; Tissue-resident memory T cells; central memory T cells; effector memory T cells; ocular redness; non-invasive tear break-up time

**Affiliation:** ^1^Laboratory of Molecular Physiology, Infection and Immunity Theme, Lee Kong Chian School of Medicine, Nanyang Technological University, 59 Nanyang Drive, Singapore 636921; ^2^Singapore Eye Research Institute, 20 College Road, Singapore 169856; ^3^Singapore National Eye Center, 11, Third Hospital Avenue, Singapore 168751; ^4^Duke-NUS Medical School, 8 College Road, 169857; ^5^Yong Loo Lin School of Medicine, 1E Kent Ridge Road Level 7, NUHS Tower Block, 119228.

***Corresponding authors:** E-mail: [gchandy@ntu.edu.sg](mailto:gchandy@ntu.edu.sg); Tel: +65 6592 3938; fax: +65 6515 0417. E-mail: [louis.tong.h.t@singhealth.com.sg](mailto:louis.tong.h.t@singhealth.com.sg); Tel: +65 9818 6221

**Figure S1. Histograms show distribution of age and gender among control and patient cohorts.**

**
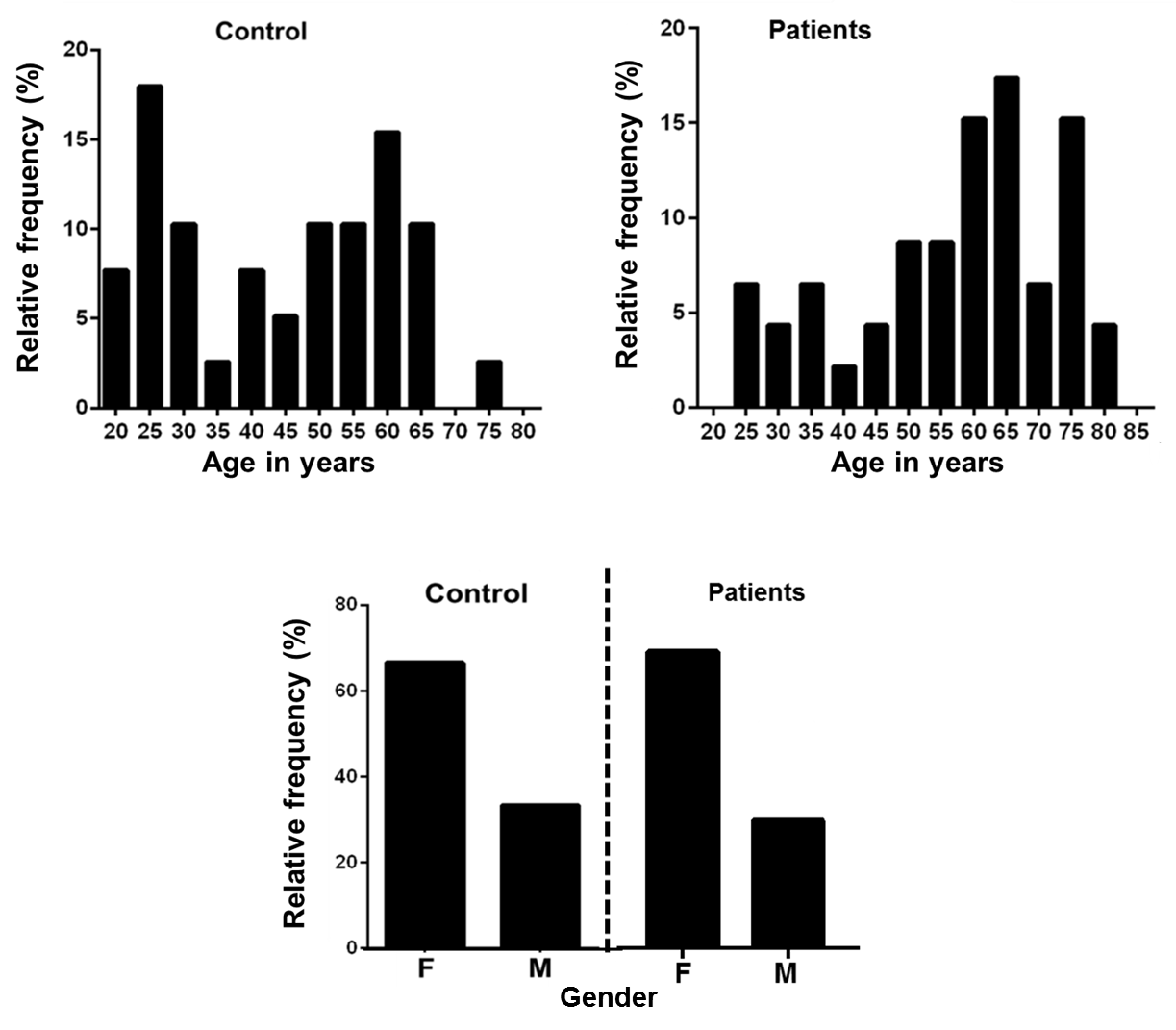
**

**Figure S2. Distribution of CXCR3 and CCR6 in control subjects. *A,*** Representative flow cytometry data of CD3^+^7-AAD^-^ live T cells showing proportions of CD4^+^ and CD8^+^ CXCR3^+^CCR6^-^, CXCR3^-^CCR6^+^, CXCR3^-^CCR6^-^ and CCR7^+^CD45RO^-^CXCR3^+^CCR6^-^subsets in healthy controls. ***B*,** Distribution of these subsets within CD4^+^ and CD8^+^ naïve, T_CM_, T_EM_ and T_EMRA_ pools in healthy controls; each data point represents a separate individual; mean ± SEM shown; R6 = CCR6, R3 = CXCR3.

**
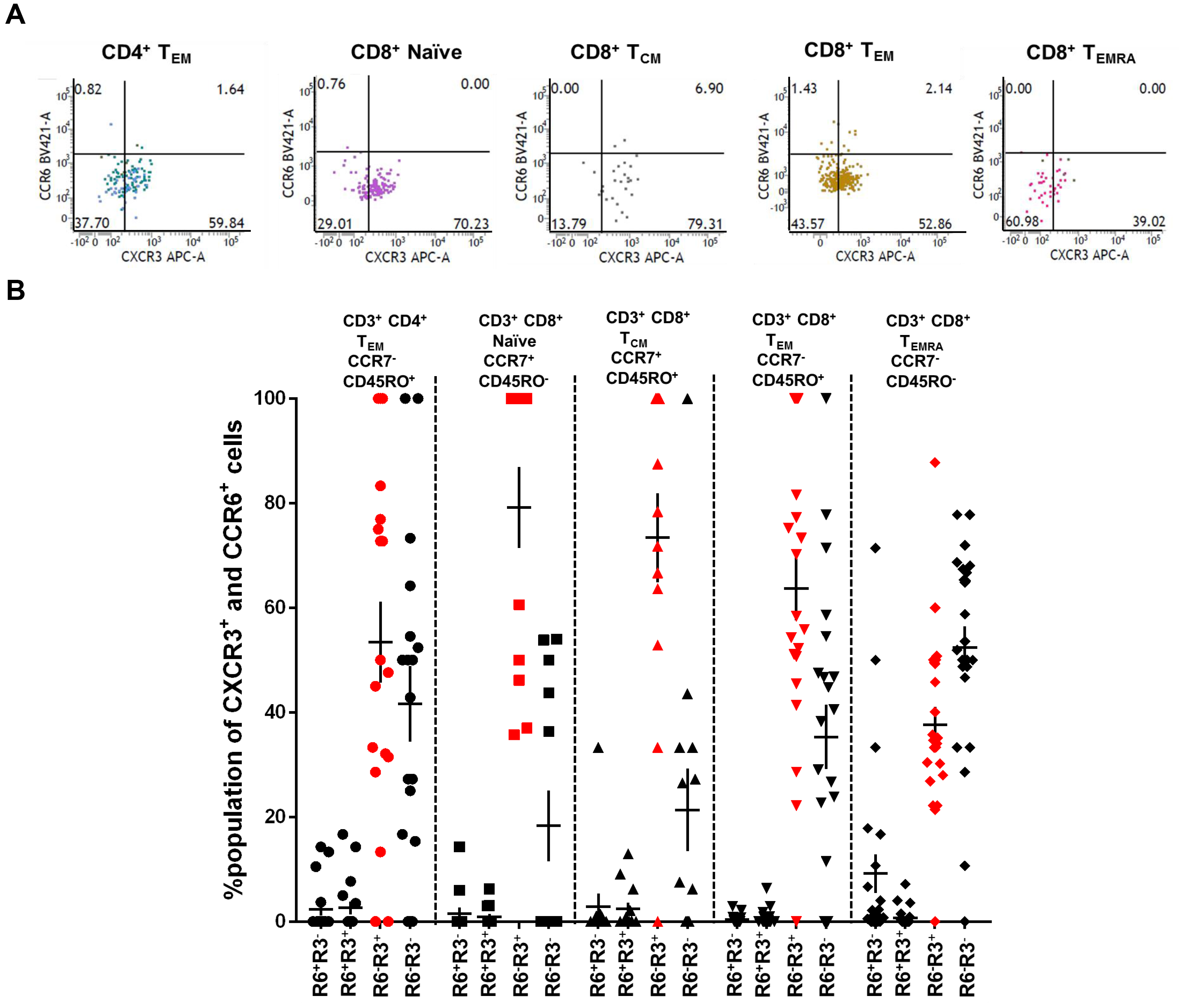
**

**Figure S3. T cell Signature-1 of Cluster-1 patients and clinical findings.**

*Upper panel:* Inverse correlation between ocular redness (OR) score and proportion of conjunctival CD8^+^ T_EM_ and CD8^+^ T_EMRA_ cells. Pearson’s correlation coefficient was analyzed. A dotted red line (OR score = 1.9) was used to distinguish controls (blue) from DED patients with immune signature 1 (red). *P* values for CD8^+^ T_EMRA_ and CD8^+^ T_EM_ are 0.002 and 0.0057. *Lower panel:* Lack of correlation between CD4^+^ and CD8^+^ T_CM_s with NI-TBUT and Schirmer’s Test.

**
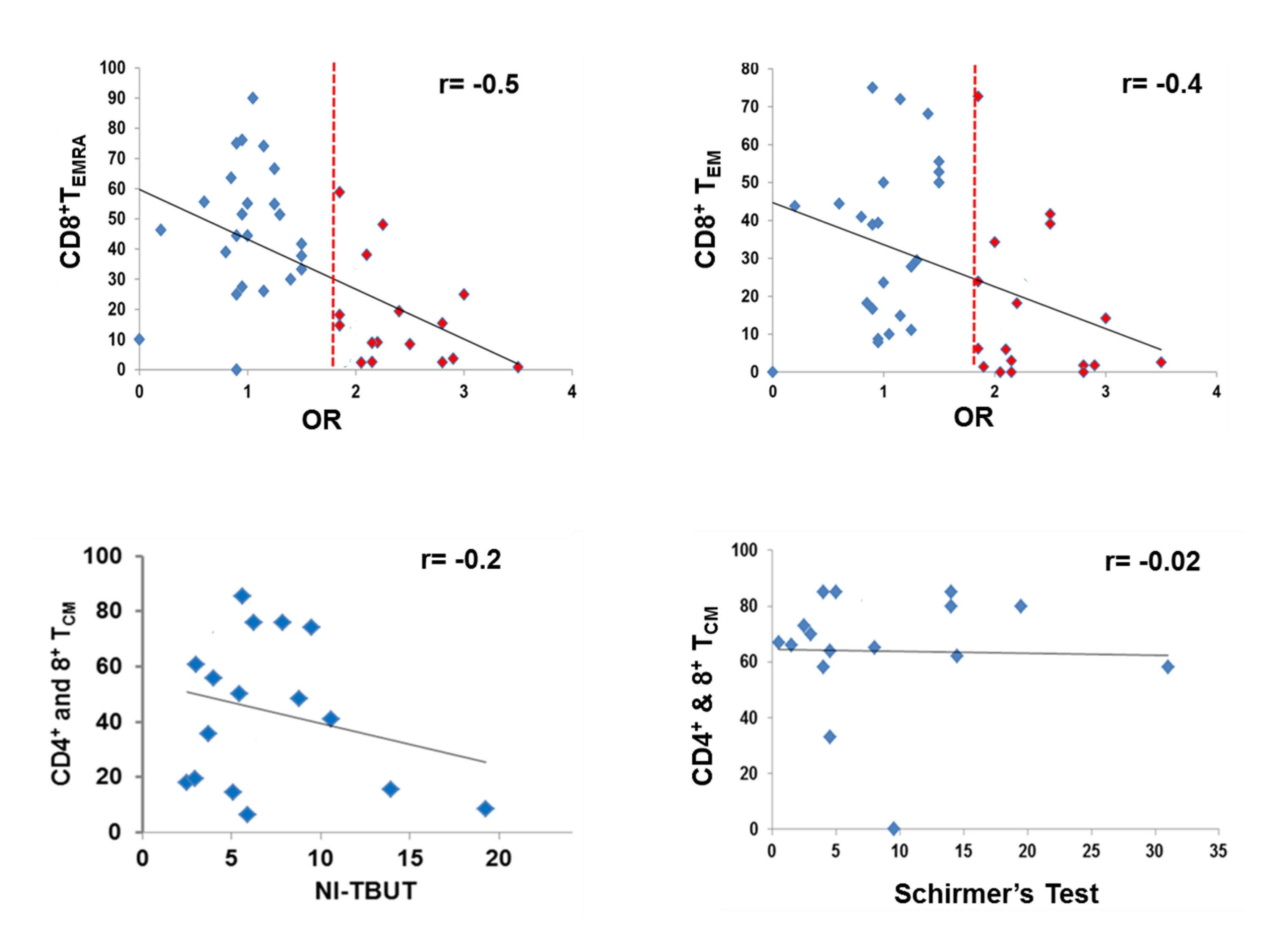
**

**Figure S4. T cell Signature-2 of Cluster-2 patients and clinical findings.**

Lack of correlation between ocular redness (OR) and Schirmer’s Test with proportions of CD4^+^ and CD8^+^ CD69^+^CD103^+^ T_EM_-T_RM_s.


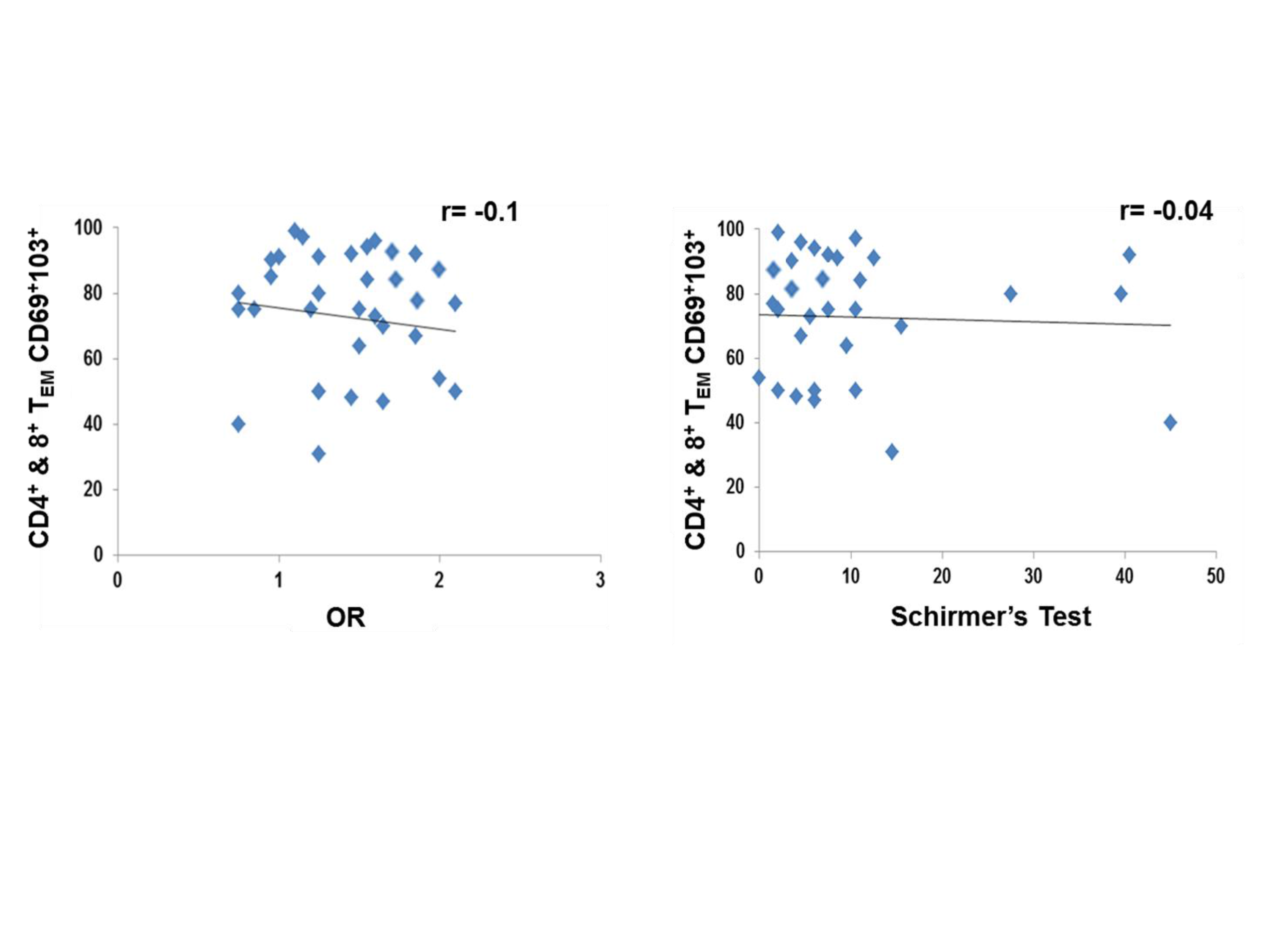


**Table S1.** Details of subjects in control population

| **Age** | ^Ɨ^**Gender** | **Ethnicity** | **OR** | **NI-TBUT** | **ST** |
| --- | --- | --- | --- | --- | --- |
| 55 | F | Chinese | 0.90 | 7.93 | 9.0 |
| 49 | F | Indian | 0.85 | 8.11 | 7.5 |
| 47 | F | Chinese | 0.90 | 5.78 | 10.0 |
| 59 | F | Chinese | 0.95 | 10.80 | 13.0 |
| 65 | F | Chinese | 0.90 | 5.55 | 13.0 |
| 55 | M | Chinese | 0.95 | 5.63 | 17.9 |
| 57 | F | Chinese | 0.60 | 8.49 | 41.5 |
| 49 | M | Chinese | 1.15 | 7.45 | 22.5 |
| 48 | F | Chinese | 1.00 | 5.08 | 5.5 |
| 64 | F | Chinese | 1.85 | 5.76 | 7.5 |
| 64 | F | Chinese | 1.50 | 9.91 | 7.5 |
| 26 | M | Malay | 0.20 | 5.74 | 7.0 |
| 29 | F | Malay | 1.00 | 10.79 | 38.5 |
| 61 | F | Chinese | 1.15 | 5.59 | 33.0 |
| 62 | M | Indian | 1.35 | 7.09 | 15.5 |
| 60 | F | Malay | 1.25 | 10.58 | 6.5 |
| 42 | F | Chinese | 1.85 | 13.99 | 9.5 |
| 27 | M | Chinese | 1.50 | 19.51 | 7.0 |
| 39 | M | Indian | 1.25 | 15.30 | 15.5 |
| 30 | M | Chinese | 1.05 | 17.44 | 27.5 |
| 25 | F | Chinese | 0.80 | 19.07 | 4.5 |
| 77 | F | Chinese | 1.50 | 17.98 | 21.0 |
| 60 | F | Chinese | 1.30 | 12.50 | 21.0 |
| 58 | M | Chinese | 1.55 | 21.52 | 20.0 |
| 32 | F | Chinese | 0.70 | 16.02 | 23.0 |
| 28 | F | Chinese | 1.15 | 11.55 | 11.5 |
| 21 | F | Chinese | 0.75 | 15.12 | 15.5 |
| 22 | M | Chinese | 1.30 | 11.37 | 21.5 |
| 22 | M | Chinese | 0.95 | 14.96 | 0.0 |
| 23 | F | Chinese | 0.90 | 10.93 | 19.5 |
| 66 | M | Caucasian | 1.40 | 13.58 | 4.5 |
| 44 | F | Chinese | 1.60 | 10.23 | 14.5 |
| 54 | F | Chinese | 1.20 | 4.68 | 9.0 |
| 25 | F | Malay | 0.55 | 6.69 | 32.5 |
| 39 | F | Indian | 0.65 | 18.64 | 32.0 |
| 24 | F | Indian | 0.65 | 7.58 | 8.5 |
| 34 | M | Indian | 1.00 | 6.80 | 43.0 |
| 26 | M | Chinese | 1.15 | 9.08 | 19.0 |
| 48 | F | Chinese | 0.90 | 9.79 | 34.5 |

OR = ocular redness

NI-TBUT = non-invasive tear breakup time (secs)

ST = Schirmer’s Test (mm)

^Ɨ^F: female, M: male

**Table S2.** Details of subjects in Cluster-1 DED patient population

| **Age** | **Gender** | **Ethnicity** | **Primary Diagnosis** | **Secondary**  **Diagnosis** | **Systemic treatment** | **OR** | **NI-TBUT** | **ST** |
| --- | --- | --- | --- | --- | --- | --- | --- | --- |
| 50 | M | Chinese | Idiopathic DED | - | - | 2.10 | 19.3 | 19.5 |
| 71 | F | Chinese | Idiopathic DED | - | - | 2.20 | 5.1 | 3.0 |
| 74 | F | Chinese | RA | Hypo-  Thyroidism;  Meibomian gland dysfunction | Hydroxy  chloroquine | 2.25 | 5.5 | 4.0 |
| 36 | M | Chinese | GvHD | Glaucoma | Mycophenolate mofetil | 3.00 | 14.0 | 4.5 |
| 27 | F | Indian | Contact lens related | - | - | 1.9 | 3.7 | 31.0 |
| 76 | F | Chinese | Idiopathic DED | - | - | 2.25 | 5.2 | 6.5 |
| 64 | M | Chinese | GvHD | - | - | 3.50 | 5.6 | 1.0 |
| 61 | F | Chinese | Myelodysplasia | - | - | 1.90 | 3.1 | 1.5 |
| 58 | F | Chinese | Idiopathic DED | - | - | 2.00 | 4.0 | 0.5 |
| 45 | F | Chinese | GvHD | Meibomian gland dysfunction | Mycophenolate mofetil | 2.90 | 9.5 | 5.0 |
| 74 | M | Chinese | Idiopathic DED | - | - | 2.00 | * | 4.5 |
| 78 | F | Chinese | Idiopathic DED | - | - | 2.15 | 2.5 | 4.0 |
| 67 | M | Chinese | Idiopathic DED | Conjunctival  chalasis | - | 2.80 | 3.1 | 14.5 |
| 74 | M | Chinese | Idiopathic DED | - | - | 2.40 | 10.6 | 14.0 |
| 82 | F | Chinese | Glaucoma eye drops (Brinzolamide) | - | - | 2.80 | 6.0 | 14.0 |
| 66 | M | Chinese | Mixed connective tissue disease | Meibomian gland dysfunction | Methotrexate | 2.05 | 6.5 | 8.0 |

DED = dry eye disease; GvHD = graft-versus-host disease; RA = rheumatoid arthritis; ST = Schirmer’s Test (mm); NI-TBUT = non-invasive tear breakup time (secs); OR = ocular redness based on average temporal bulbar redness.

^Ɨ^ Includes dry eye related to post-menopausal changes and mild Meibomian gland dysfunction;

*: difficulty opening eyelids.

**Table S3.** Details of subjects in Cluster-2 DED patient population

| **Age** | **Gender** | **Ethnicity** | **Primary diagnosis** | **Secondary diagnosis** | **Systemic treatment** | **OR** | **NI-TBUT** | **ST** |
| --- | --- | --- | --- | --- | --- | --- | --- | --- |
| 60 | M | Chinese | ^Ɨ^ Idiopathic DED | - | - | 1.95 | 7.9 | 2.5 |
| 75 | F | Chinese | Idiopathic DED | SLE; Thyroid Carcinoma | Hydroxy  chloroquine | 2.50 | 5.9 | 6.0 |
| 64 | F | Chinese | **^Ɨ^**Idiopathic DED |  |  | 1.5 | 5.93 | 9.5 |
| 49 | F | Chinese | SLE |  | Prednisolone, Colchicine | 1.00 | 5.33 | 8.5 |
| 44 | M | Malay | Idiopathic DED | - | - | 1.95 | 7.8 | 9.5 |
| 24 | F | Chinese | Idiopathic DED | Allergic Conjunctivitis |  | 1.25 | 2.58 | 39.5 |
| 29 | M | Chinese | Idiopathic DED | Vernal Conjunctivitis |  | 1.45 | 4.40 | 40.5 |
| 64 | F | Malay | Idiopathic DED |  |  | 1.15 | 3.47 | 10.5 |
| 45 | F | Chinese | GvHD | Meibomian gland dysfunction | - | 3.45 | 9.50 | 5.00 |
| 74 | F | Malay | Idiopathic DED |  |  | 1.2 | 7.11 | 7.5 |
| 53 | F | Indian | Idiopathic DED |  |  | 0.85 | 7.37 | 10.5 |
| 61 | F | Chinese | RA |  |  | 1.85 | 5.35 | 7.5 |
| 23 | F | Chinese | Idiopathic DED |  |  | 0.75 | 6.31 | 45.0 |
| 58 | F | Chinese | Idiopathic DED |  |  | 1.85 | 6.22 | 4.5 |
| 29 | M | Chinese | Idiopathic DED |  |  | 0.75 | 10.55 | 2.0 |
| 67 | F | Chinese | Idiopathic DED |  |  | 1.5 | 5.55 | 2.0 |
| 73 | F | Chinese | Idiopathic DED |  |  | 1.95 | * | 0.0 |
| 36 | F | Chinese | **^ƗƗ^**Sjogren’s syndrome |  | Prednisolone | 1.1 | 3.35 | 2.0 |
| 65 | F | Chinese | Sjogren’s syndrome |  |  | 1.6 | 5.07 | 5.5 |
| 56 | M | Chinese | Idiopathic DED |  |  | 1.6 | 5.29 | 4.5 |
| 35 | M | Chinese | GvHD | - | - | 2.15 | 3.5 | 0.0 |
| 54 | F | Chinese | Sjogren’s syndrome |  | Methotrexate | 1.6 | 4.11 | 6.0 |
| 63 | F | Chinese | Idiopathic DED |  |  | 1.2 | 3.73 | 10.5 |
| 74 | F | Chinese | RA |  | Hydroxy  chloroquine | 2.1 | 2.87 | 1.5 |
| 51 | F | Caucasian | Sjogren’s syndrome |  |  | 0.9 | 6.21 | 3.5 |
| 41 | F | Malay | Idiopathic DED |  |  | 0.9 | 3.28 | ND |
| 59 | F | Chinese | Sjogren’s syndrome |  |  | 1.2 | 2.59 | 12.5 |
| 72 | F | Malay | Glaucoma eyedrops (Latanoprost) |  |  | 1.5 | 2.97 | 11.0 |
| 52 | F | Chinese | SLE |  | Hydroxy  chloroquine | 2.1 | 6.02 | 2.0 |
| 62 | F | Malay | Idiopathic DED |  |  | 1.2 | 3.63 | 6.0 |
| 56 | F | Indian | Idiopathic DED | GvHD,  **^ƗƗƗ^**post - LASIK CMV viremia |  | 0.9 | 3.73 | 1.0 |
| 68 | F | Indian | Idiopathic DED |  |  | 1.5 | 5.00 | 6.0 |
| 51 | F | Indian | Idiopathic DED |  |  | 0.7 | 5.55 | 27.5 |
| 75 | F | Chinese | RA |  | Hydroxychloroquine, Methotrexate, Sulfasalazine | 1.6 | 3.4 | 5.5 |
| 65 | F | Chinese | Idiopathic DED |  |  | 1.4 | 2.61 | ND |
| 91 | F | Chinese | Glaucoma eyedrops (Timolol) |  |  | 1.2 | 3.44 | 4.0 |

**^Ɨ^** Includes dry eye related to post-menopausal changes and mild Meibomian gland dysfunction;

*: difficulty opening eyelids; **^ƗƗ^** Primary Sjogren’s syndrome; **^ƗƗƗ^** LASIK = Laser assisted in-situ keratomileusis: tears not collected. ST = Schirmer’s Test (mm); NI-TBUT = non-invasive tear breakup time (secs); OR = ocular redness based on average temporal bulbar redness; SLE = systemic lupus erythematosus.

**Table S4.** Absolute numbers of T cell subsets in control and in Cluster-1 and Cluster-2

|  | Control | Cluster-1 | Cluster-2 | *P* values |
| --- | --- | --- | --- | --- |
| **CD3^+^ Naïve** | 58 ± 12 | 180 ± 10 | 44 ± 10 | *P*<0.0001 |
| CD4^+^ Naïve | 6 ± 2 | 20 ± 2 | 4 ± 2 |  |
| CD8^+^ Naïve | 52 ± 10 | 160 ± 7 | 40 ± 8 |  |
| **CD3^+^ T_CM_** | 73 ± 12 | 588 ± 73 | 66 ± 8 | *P*<0.0001 |
| CD4^+^ T_CM_ | 29 ± 2 | 220 ± 23 | 32 ± 3 |  |
| CD8^+^ T_CM_ | 44 ± 10 | 368 ± 40 | 34 ± 5 |  |
| **CD3^+^ T_EM_** | 632 ± 12 | 176 ± 10 | 853 ± 10 | *P*<0.0001 |
| CD4^+^ T_EM_ | 118 ± 4 | 29 ± 2 | 265 ± 2 |  |
| CD8^+^ T_EM_ | 514 ± 8 | 147 ± 8 | 588 ± 8 |  |
| **CD3^+^ T_EMRA_** | 691 ± 12 | 162 ± 10 | 617 ± 12 | *P*<0.0001 |
| CD4^+^ T_EMRA_ | 29 ± 2 | 15 ± 2 | 28 ± 2 |  |
| CD8^+^ T_EMRA_ | 662 ± 10 | 147 ± 8 | 589 ± 10 |  |

**Table S5.** Absolute numbers of T_EM_ and T_EMRA_ cells in control and Cluster-2

|  | Control | Cluster-2 | *P* values |
| --- | --- | --- | --- |
| **CD8^+^ T_EMRA_** | 662 ± 10 | 589 ± 12 |  |
| CD8^+^T_EMRA_-T_RM_ (CD69^+^CD103^-^) | 132 ± 2 | 41 ± 2 | *P* <0.05 |
| CD8^+^T_EMRA_-T_RM_ (CD69^+^CD103^+^) | 251 ± 6 | 528 ± 8 | *P* <0.0001 |
| CD8^+^T_EMRA_-T_RCM_ (CD69^-^CD103^+^) | 146 ± 2 | 16 ± 2 | *P* <0.01 |
| CD8^+^T_EMRA_  CD69^-^CD103^-^ | 133 ± 0 | 4 ± 0 |  |
| **CD4^+^T_EM_** | 118 ± 4 | 265 ± 2 |  |
| CD4^+^T_EM_-T_RM_ (CD69^+^CD103^-^) | 2 ± 0 | 27 ± 0 |  |
| CD4^+^T_EM_-T_RM_ (CD69^+^CD103^+^) | 92 ± 1 | 106 ± 1 |  |
| CD4^+^T_EM_-T_RCM_ (CD69^-^CD103^+^) | 9 ± 2 | 101 ± 1 | *P* <0.01 |
| CD4^+^T_EM_  CD69^-^CD103^-^ | 15 ± 1 | 31 ± 0 |  |
